# Abnormal myocardial perfusion pattern in convalescent Kawasaki Disease patients assessed by stress perfusion cardiovascular magnetic resonance

**DOI:** 10.1186/1532-429X-16-S1-P216

**Published:** 2014-01-16

**Authors:** Konstantinos Bratis, Amedeo Chiribiri, Tarique Hussain, Thomas Krasemann, Markus Henningsson, Alkystis Phinikaridou, Rene Botnar, Sophie Mavrogeni, Eike Nagel, Gerald F Greil

**Affiliations:** 1 Cardiovascular Imaging, King's College London, London, UK; 2 A' Cardiology, Onassis Cardiac Surgery Centre, Athens, Greece; 3 Pediatric Cardioloy, King's College London, London, UK

## Background

Kawasaki Disease is a generalised systemic vasculitis involving blood vessels throughout the body, although the coronary arteries virtually always are involved. Previous evidence from echocardiographic, Single Photon Emission Computed Tomography (SPECT) and Positron Emission Tomography (PET) studies of myocardial blood flow and flow reserve in KD patients revealed reduced hyperemic flows and flow reserve, suggestive of an impaired vasodilatory capacity. The aim of the study was to determine whether myocardial blood flow and flow reserve derived from perfusion Cardiac Magnetic Resonance Imaging (CMR), is impaired in children with a previous history of KD.

## Methods

Patients with known chronic KD and coronary involvement (mean age: 10.2 ± 7.2 years/mean duration from KD acute illness to CMR exam: 4.95 ± 5.6 years) underwent functional imaging, first-pass stress/rest perfusion, Late Gadolinium Enhancement (LGE) and Magnetic Resonance Angiography (MRA). All exams were analysed visually and quantitatively. Perfusion myocardial reserve was assessed by semi- quantitative methods (Mean Perfusion Reserve Index- MPRI). Mean per patient and segmental results were derived. Results were correlated to the epicardial vessels patency.

## Results

**Figure 1:**
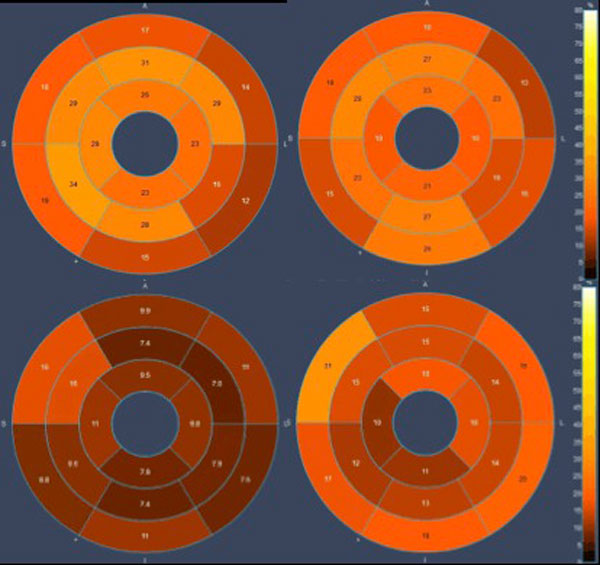
**Polar map presentation of signal intensity relative upslope at stress (left) and at rest (right), in a patient with regressed CALs (upper row), and persistent CALs (Giant aneurysm of LMCA 17 × 17 × 20 mm/Aneurysm of LAD origin 4 × 4 mm/Dilation od distal RCA 2. 8 × 8 mm) (lower row)**. Diffuse significantly impaired myocardial perfusion reserve is revealed in both cases. Additional extensive regional perfusion impairment of the basal to apical anterolateral and inferolateral segments, corresponding to associated diseased epicardial arteries, is demonstrated in the second case. CAL: Coronary Artery Lesion

Fourteen patients with known chronic KD underwent the CMR protocol (mean age: 10.2 ± 7.2 years/mean duration from KD acute illness to CMR exam: 4.95 ± 5.6 years). 5/14 patients presented with persisting epicardial lesions in MRA. 1/14 patient presented inducible perfusion defect. All patients had significantly impaired mean perfusion reserve index compared to normal adult subjects (0.866 ± 0.256 vs 2.46 ± 0.3, p < 0,001). Perfusion reserve impairment concerned all segments analysed, irrespective of the coronary artery status involved. There was no statistical difference in mean MPRI between patients without and with persisting CALs (1.01 ± 0.32 vs 0,792 ± 0,225, p: 0,31). (Figure 1) Patient's clinical and CMR characteristics are provided in Table 1.

**Table 1:** CMR Results of the 14 study patients.

Patient No	Age (y)	Gender	Diagnosis of Kawasaki Disease	Duration of Disease	EF (%)	Coronary Artery Status	Treatment	MRA	First- pass Perfusion Stress/Rest	Mean MPRI	Wall Motion Abnormality	LGE	Exam under Anaesthesia
1	3	M	Complete	1 y 5 m	57	II	IVIG, Aspirin	Dilation of RCA origin 3 mm/Dilation of LAD- LCX bifurcation 4 mm	Normal/Normal	1.05	No	No	Yes

2	7	M	Complete	2 y 5 m	62	II	IVIG, Aspirin	Dilation of LM 4 mm	Normal/Normal	1.01	No	No	Yes

3	8	F	Complete	1 y 5 m	57	I	IVIG, Aspirin	Normal	Normal/Normal	1.62	No	No	Yes

4	8	M	Complete	9 m	54	I	IVIG, Aspirin	Normal	Normal/Normal	0.95	No	No	No

5	5	M	Complete	6 m	65	I	IVIG, Aspirin	Normal	Normal/Normal	0.7	No	No	Yes

6	19	M	Complete	7 y	63	I	IVIG, Aspirin	Normal	Normal/Normal	1.06	No	No	No

7	8	F	Complete	3 y	65	II	IVIG, Aspirin	Dilation of LAD origin 3.3 mm	Normal/Normal	0.66	No	No	Yes

8	3	F	Complete	1 y	71	I	IVIG, Aspirin	Normal	Normal/Normal	0.48	No	No	Yes

9	10	M	Complete	4 y	61	I	IVIG, Aspirin	Normal	Normal/Normal	0.98	No	No	Yes

10	17	F	Complete	14 y 9 m	59	II	IVIG, Aspirin	Dilation of LM/LAD 3.5 mm	Normal/Normal	0.54	No	No	No

11	27	M	Complete	14 y	60	I	IVIG, Aspirin	Normal	Normal/Normal	1.3	No	No	No

12	6	F	Complete	3 y	57	I	IVIG, Aspirin	Normal	Normal/Normal	1.01	No	No	No

13	18	M	Complete	16 y	55	I	IVIG, Aspirin	Normal	Posterior defect at Stress and Rest	1	Posterior wall akinesis	Posterior	No

14	4	F	Complete	3 m	64	II	IVIG, Aspirin	Giant anevrysm of LMCA 17*17*20 mm/Anevrysm of LAD origin 4*4 mm/Dilation of distal RCA 2.8*8 mm	Basal, mid ventricular and apical anterolateral and infero lateral stress induced perfusion defect	0.7	No	No	Yes

Group I: regressed CALs, Group II: persistent CALs CMR: Cardiac Magnetic Resonance, CAL: Coronary Artery Lession, EF: Ejection Fraction, MRA: Magnetic Resonance Angiography, LGE: Late Gadolinium Enhancement, M: male, F: Female, LAD: Left Anterior Descending Coronary Artery, LCx: Circumflex Coronary Artery, RCA: Right Coronary Artery, IVIG: Intravenous Immunoglobulin

## Conclusions

First-pass perfusion CMR identifies abnormal perfusion reserve in KD convalescent patients. Impaired MPRI reflects coronary microvascular dysfunction. Visual assessment failed to reveal the abnormal myocardial perfusion pattern. Quantitative CMR perfusion analysis, compared to visual assessment, influences substantially the appreciation of myocardial perfusion pattern in Kawasaki Disease.

## Funding

The authors acknowledge financial support from the Department of Health through the National Institute for Health Research (NIHR) comprehensive Biomedical Research Centre award to Guy's & St Thomas' NHS Foundation Trust in partnership with King's College London and King's College Hospital NHS Foundation Trust. The Division of Imaging Sciences receives also support as the Centre of Excellence in Medical Engineering (funded by the Welcome Trust and EPSRC; grant number WT 088641/Z/09/Z) as well as the BHF Centre of Excellence (British Heart Foundation award RE/08/03). Dr Bratis acknowledge receiving training grant by the Hellenic Society of Cardiology. The rest of the authors have no financial activities related to the present article to disclose.

